# Supplementary material for: Time-Resolved X-ray Observation of Intracellular Crystallized Protein in Living Animal
Source: Int J Mol Sci. 2023 Nov 29;24(23):16914. doi: 10.3390/ijms242316914 (PMC10706802; doi:10.3390/ijms242316914)
Supplement: Supplementary file 1 [file ijms-24-16914-s001.zip › ijms-2719529-supplementary.pdf]

Supplementary materials for

Time-Resolved X-ray Observation of Intracellular Crystallized Protein in Living Animal

Masahiro Kuramochi [1,*,†], Ibuki Sugawara [1,†], Yoichi Shinkai [2], Kazuhiro Mio [3] and Yuji C. Sasaki [4]

[1] Graduate School of Science and Engineering, Ibaraki University, Hitachi 316-8511, Japan
[2] Molecular Neurobiology Research Group, Biomedical Research Institute, National Institute of Advance Industrial Science and Technology (AIST), Tsukuba 305-8566, Japan
[3] AIST-UTokyo Advanced Operando-Measurement Technology Open Innovation Laboratory (OPERANDO-OIL), National Institute of Advanced Industrial Science and Technology (AIST), Kashiwa 277-8565, Japan
[4] Graduate School of Frontier Sciences, The University of Tokyo, Kashiwa 277-8561, Japan
* Correspondence: masahiro.kuramochi.vw26@vc.ibaraki.ac.jp
† These authors contributed equally to this work.

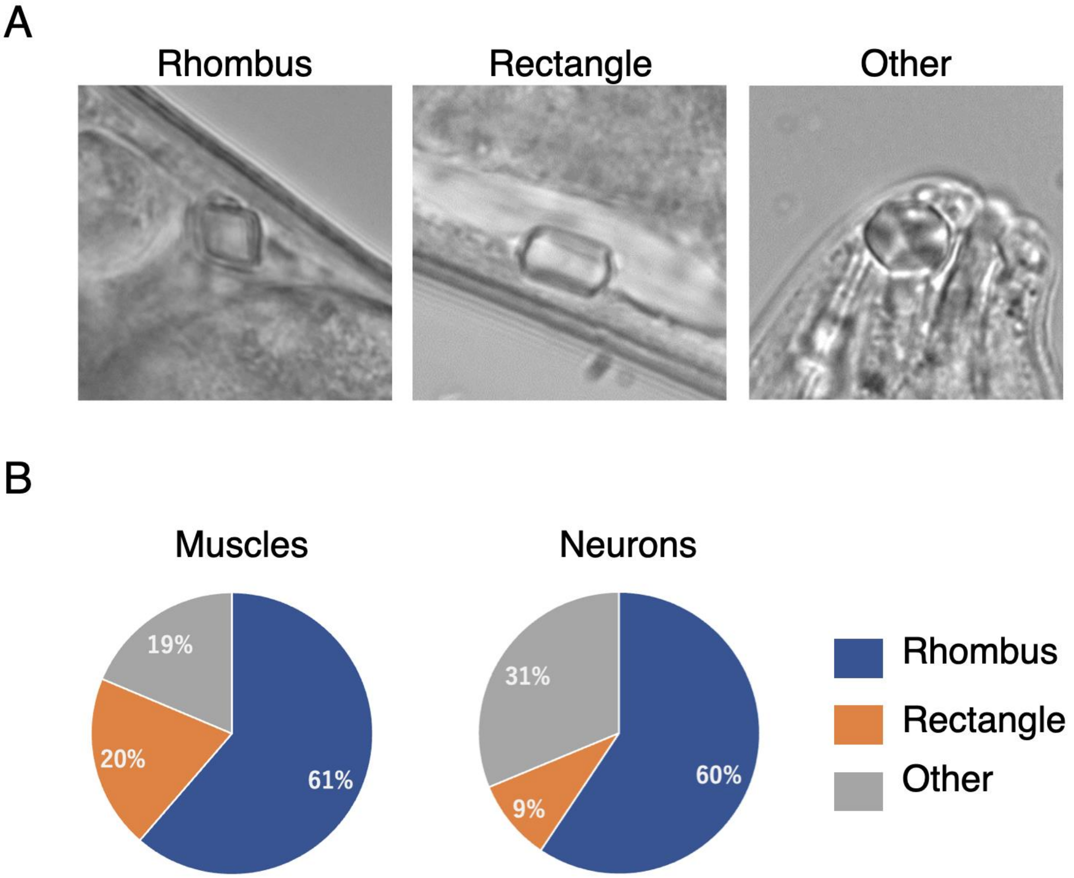

Figure S1. Classification of the Xpa molecular aggregates based on shape. (A) Microscopy images of the aggregate shapes. These shapes can be classified into three categories: rhombus, rectangle, and other. (B) Proportion of the rhombus, rectangle, and other aggregates in the muscles and neurons.

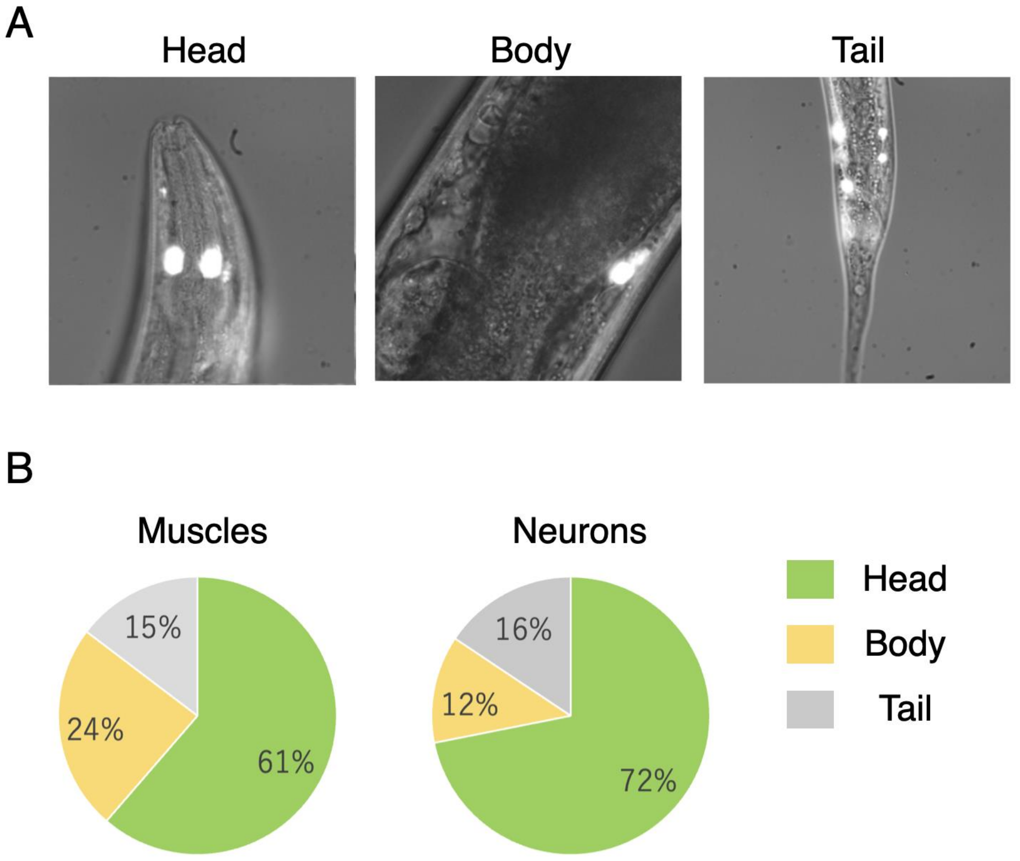

Figure S2. Sites of the Xpa molecular aggregates expression in *C. elegans* cells. (A) Images of the Xpa aggregation expressed in the head, body and tail. (B) Proportion of the aggregate expression sites in the muscles and neurons.

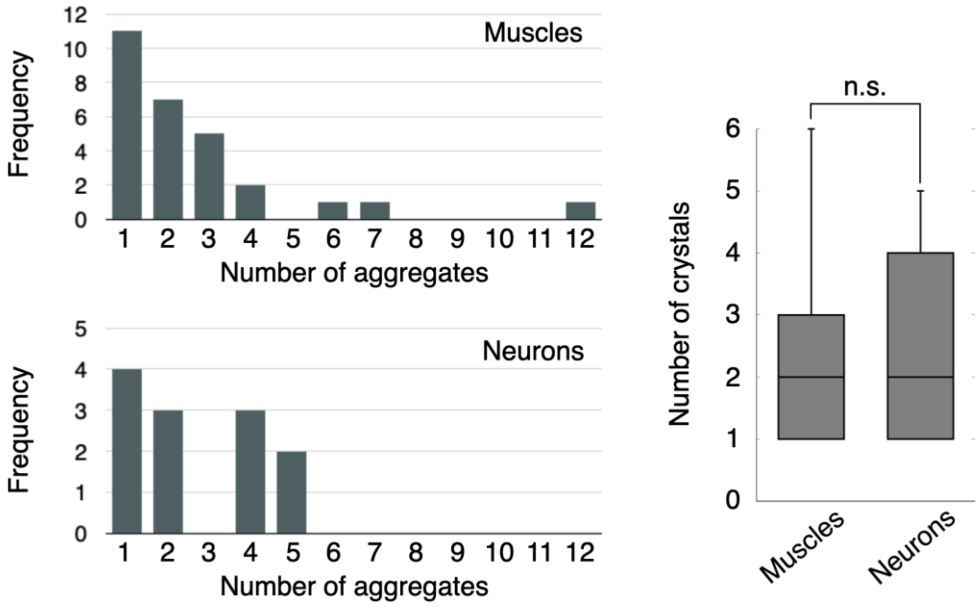

Figure S3. Distribution of the number of aggregates per single animal. Histogram (left) and boxplot (right) in muscles and neurons. Wilcoxn rank sum test is performed to compare muscles and neurons. 'n.s.' indicates no significance.

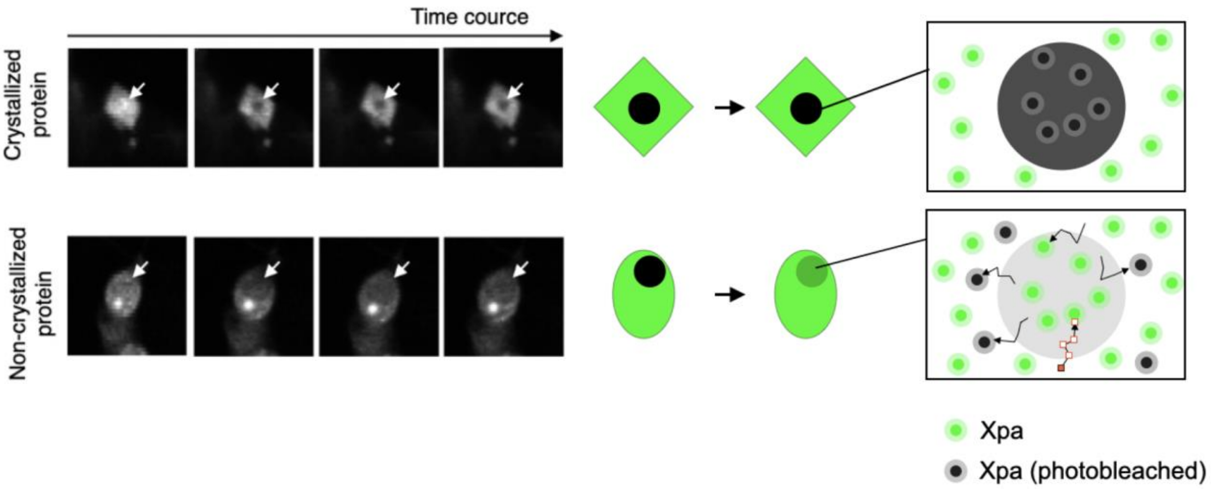

Figure S4. Summary of FRAP measurement results

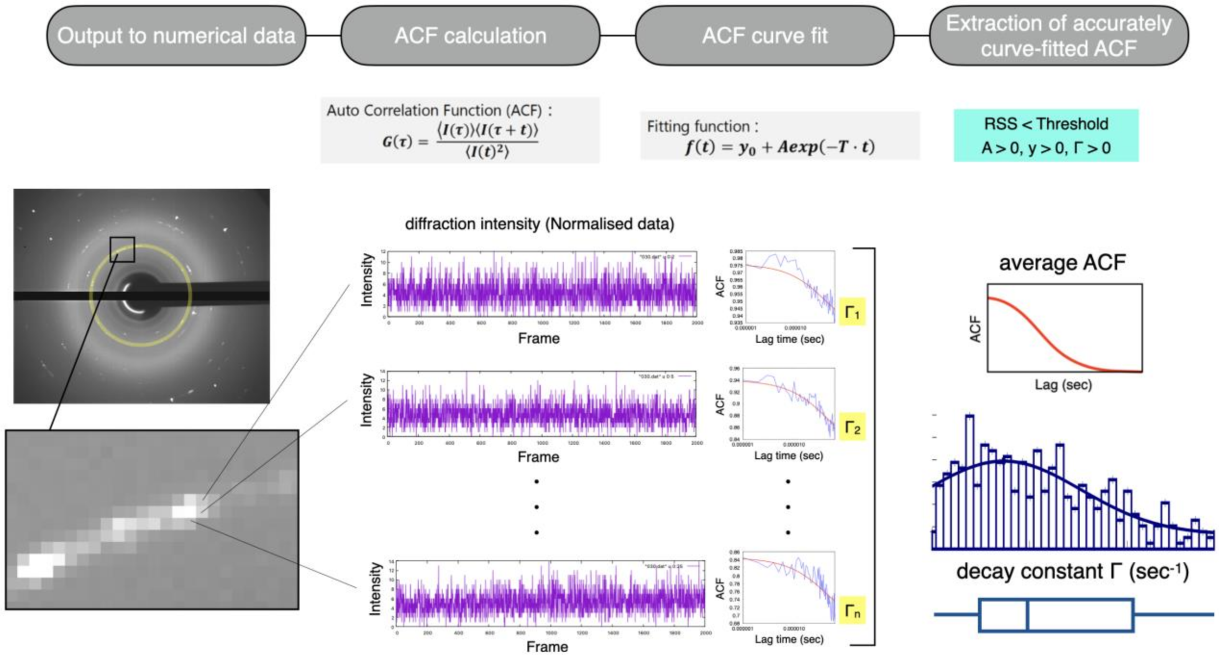

Figure S5. Schematic of the autocorrelation for the DXB data. The region of interest (ROI) is selected from the Debye–Scherrer ring. Time autocorrelation is calculated from the intensity fluctuations of a single pixel after correcting for long-term intensity changes. The single-pixel ACF is fitted with a single exponential curve. To exclude inaccurate ACF curves, precise ACF curves are individually selected based on various parameters and the residual sum of squares (RSSs) from the fitted model. The selected single-pixel ACFs are used to assess the distribution and average ACF.

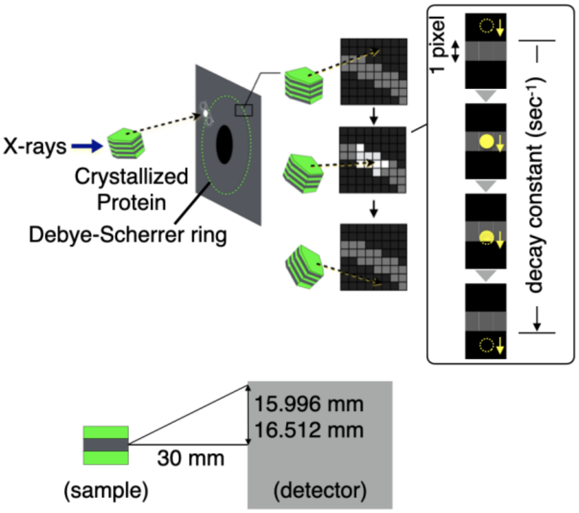

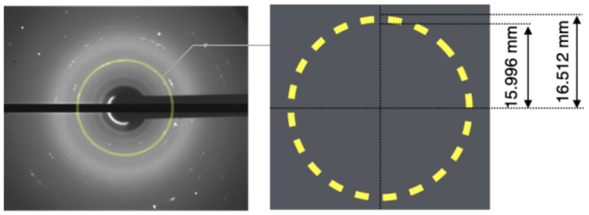

Angle	2θ [deg]	2θ [rad]	d-space (nm)	decay constant (sec^{-1})
Low	28.067	0.489	0.318	0.0185 (50°C)
High	28.828	0.503	0.309	

	Rotational diffusion	
	2θ (rad^2/sec)	d-space ($nm^2/$
Low	1.106E-03	4.677E-04
High	1.170E-03	1.170E-03

Figure S6. Rotational diffusion coefficient of the Xpa crystals. Schematic of the diffraction spot movement in DXB. The angular width and d-space are computed from the diffraction signal position and camera length. The rotational diffusion coefficient are determined using this information along with the water reduction constant.
